# Characteristics of personal health information management groups: findings from an online survey using Amazon’s mTurk

**DOI:** 10.5195/jmla.2017.312

**Published:** 2017-10-01

**Authors:** Sujin Kim, Jeffrey T. Huber

## Abstract

**Objective:**

The study characterized three groups with different levels of familiarity with personal health information management (PHIM) in terms of their demographics, health knowledge, technological competency, and information sources and barriers. In addition, the authors examined differences among PHIM groups in subjective self-ratings and objective test scores for health literacy.

**Methods:**

A total of 202 survey participants were recruited using Amazon’s Mechanical Turk (mTurk) service, a crowdsourcing Internet service. Using K-means clustering, three groups with differing levels of familiarity with PHIM were formed: Advanced, Intermediate, and Basic.

**Results:**

The Advanced group was the youngest, and the Basic group contained the highest proportion of males, whereas the Intermediate group was the oldest and contained the fewest males. The Advanced group was significantly more likely to engage in provider- or hospital-initiated PHIM activities such as emailing with providers, viewing test results online, and receiving summaries of hospital visits via email or websites than the other groups. The Basic group had significantly lower information management skills and Internet use than the other groups. Advanced and Basic groups reported significant differences in several information barriers. While the Advanced group self-reported the highest general literacy, they scored lowest on an objective health literacy test.

**Conclusions:**

For effective personal health records management, it is critical to understand individual differences in PHIM using a comprehensive measure designed to assess personal health records–specific activities. Because they are trained to perform an array of information management activities, medical librarians or patient educators are well positioned to promote the effective use of personal health records by health consumers.

## INTRODUCTION

Personal health information management (PHIM) refers to both the practices and activities that individuals perform to collect, organize, find or refind, use, and share personal health information needed to fulfill health-related tasks and roles [1, 2]. These activities involve an entire spectrum of information management activities that have been conventionally performed by well-trained, experienced information professionals, such as librarians or archivists, for large collections and diverse patron groups. With the advance of consumer health technology and the patient empowerment movement, health consumers are now being asked to perform these challenging activities despite their inexperience.

Making the situation more difficult, PHIM is a special case of personal information management that is associated with multiple interactions among varying users (e.g., patients, providers, insurance companies); complex health information and systems (e.g., labs, medications, insurance); and advanced health information technology tools (e.g., personal health records, personal health devices) [3–5].

Various consumer health technologies claim to facilitate consumers’ information management, but in reality, these technologies are still in their infancy stage [6, 7]. For example, personal health records (PHRs), as hubs of health care–related documents, are thought to be a promising way to promote patients’ engagement in their health care. Whereas PHIM refers to the activities and processes that patients use to manage their health information, PHRs represent a web technology solution to PHIM. Despite the growing PHR adoption rate in major hospitals, not all features are available or utilized. Patients’ favored features of PHRs include scheduling appointments, messaging securely with health care providers, and requesting prescription refills [8–10]. Underutilized features include viewing laboratory test results, updating health conditions, reading educational materials, and synchronizing external health devices and cell phone applications for monitoring health-related activities [11].

Unlike professionally controlled medical records or health information, the successful use of patient-centered PHRs is closely related to an individual’s level of health literacy. Lester and colleagues found that patients’ understanding of medical records and legal liability are barriers to successful PHIM and PHR use [12]. Previous studies also reported that demographic characteristics of individuals with limited health literacy—including being elderly, chronically ill, an ethnic minority, or male or having a low socioeconomic status or education level—are somewhat related to poor PHIM practices [13, 14]. However, these results are limited and inconclusive due to small sample sizes, restricting their generalizability and ability to accurately characterize individual variations in PHIM.

Over the past years, literacy scales have been developed to assess individuals’ health literacy levels, but none have focused on health literacy in the context of PHIM. Conventional health literacy assessments, such as the Rapid Estimate of Adult Literacy in Medicine and the Test of Functional Health Literacy in Adults, do not adequately measure PHIM skills because they do not assess technology skills or diverse information management activities [15]. Moreover, these conventional scales are mostly based on self-reported surveys that ask how much respondents know or are aware of given information or technologies. Therefore, their relationship to objective measurements, such as direct test scores, is not known. Most importantly, conventional health literacy scales do not distinguish between different aspects of health literacy or highlight areas for further improvement.

In early electronic health record (EHR) system design and adoption, medical librarians actively engaged in providing custom links to the medical literature to meet individual patient needs. Similar to EHR systems, librarians in large academic hospitals noted that PHRs could be used as a new vehicle for delivering quality health information and could be a focus of library instruction [16]. Indeed, a role of librarians in teaching best practices in PHIM is critical, as Tang and colleagues identified lack of knowledge of PHRs as a major barrier to successful PHIM [11].

Although the body of literature on health literacy and PHR systems has grown considerably over the last decade [11], little research has been conducted to characterize different levels PHIM activities and their relationship to health literacy [17]. Therefore, the main objective of this study was to characterize different PHIM groups in terms of their demographics, health knowledge, technology competency, and information sources and barriers.

## METHODS

### Participants

The authors recruited participants using a relatively low incentive via Amazon’s Mechanical Turk (mTurk) service, a crowdsourcing Internet service in which individuals participate in surveys or subject-tagging activities that require human intelligence. Despite some concerns about recruiting subjects using mTurk or other crowd-sourcing services [18], previous studies support the use of the mTurk service as a valid survey research method that produces results similar to other reliable recruitment methods [19–23]. Using the mTurk service, we assumed that participants were US residents (based on ownership of a US bank account), had at least a 95% task approval rate for their previous assignments, and were able to complete the survey in English. Non-US residents were excluded because our measures might not be valid for non-English-speaking, non-US participant samples. The initial survey participants received a $1.50 incentive. These respondents were then asked to participate in an additional test for another $1.50 incentive.

### Measures

We first administered a PHIM survey composed of questions about demographics (e.g., age, gender, ethnicity, education, and income), health knowledge and technology competency, utilization of information sources, information barriers (e.g., cost, knowledge, language), and self-reported health literacy. In addition, 9 items measuring the participant’s familiarity with PHIM activities were included: (1) making appointments with a provider by email or on a website, (2) seeing a provider use a computer or handheld device to look up test results or other information, (3) emailing a provider’s office and receiving an answer to a medical question, (4) looking for test results on a website supplied by a provider, (5) receiving a summary of hospital visits by email or on a website, (6) reading hospital websites about health-related information, (7) requesting copies of medical records, (8) searching websites to find answers to health-related questions, and (9) posting or answering health-related questions on websites.

The overall reliability coefficient (Cronbach’s alpha) for this set of 9 PHIM activity–related questions was 0.849, which indicates a high level of internal consistency in this study sample. A total of 202 responses were included for analysis after responses that were from duplicate Internet protocol (IP) addresses and/or were missing answers to more than 25% of the questions were excluded.

For those who completed the initial PHIM survey, we then administered a Research Triangle Initiative (RTI) health literacy test [24, 25], which is composed of 25 items grouped into 5 subscales for different health literacy areas: (1) identifying and understanding health-related text (i.e., print-prose); (2) interpreting information and/or data in the form of tables, charts, pictures, symbols, maps, and videos (i.e., print-document); (3) completing computations (i.e., print-quantitative); (4) making inferences based on the information presented or applying information to a specific scenario (i.e., oral); and (5) utilizing the Internet/computer to obtain health information (i.e., Internet). Although the RTI test was not designed to measure PHIM literacy specifically, it measures multiple aspects of health literacy. A total of 139 respondents (68.8% of the initial survey sample) participated in the RTI test.

Except for demographic data on age, gender, and ethnicity, study variables were measured using Likert scales ranging from 1 (“strongly disagree” or “unsatisfactory”) to 7 (“strongly agree” or “highly satisfactory”). For education and income variables, we used numerical scales representing 7 education levels: 1: middle school graduate (or equivalent), 2: high school graduate (or equivalent), 3: some college (1–4 years, no degree), 4: associate’s degree, 5: bachelor’s degree (BA, BS, etc.), 6: master’s degree (MA, MS, etc.), and 7: professional or doctorate degree (MD, JD, PhD, etc.); and 7 income levels: 1: Less than $9,999, 2: $10,000–$24,999, 3: $25,000–$49,999, 4: $50,000–$69,999, 5: $70,000–$99,999, 6: $125,000–$149,999, and 7: more than $150,000.

### Data analysis

The K-means clustering technique was used to identify three groups of individuals with different levels of the nine PHIM activities. Clustering began with the construction of initial PHIM group centers to assign individuals to a predefined number of clusters based on the distance of the nine PHIM activities from each of the cluster centers. The locations of cluster centers were updated based on the mean values of cases in each cluster until any reassignment of cases made the clusters more internally variable. We predefined three cluster memberships representing Advanced, Intermediate, and Basic levels of PHIM activities based on previous health literacy assessments [26, 27].

After individuals were assigned to one of the three groups via clustering, we tested for differences among the three groups using analysis of variance (ANOVA) and Tukey post-hoc analyses. Explanatory variables for the group differences included demographics (e.g., age, gender, ethnicity, education, and income); health knowledge (e.g., health care experience, content knowledge, and health care familiarity); technology competency (e.g., general computing, information management, and Internet use); utilization of information sources (e.g., health care professionals, family, friends, insurance, pharmacy, websites, schools, libraries, and government); and information barriers (e.g., financial issues, knowledge, language, human resources, culture, and superfluous information). We also performed Pearson’s correlations between RTI test scores and self-reported literacy ratings across the three PHIM groups.

## RESULTS

Tables 1, 2, and 3 provide complete lists of and details about variables.

**Table 1:** Demographic characteristics and personal health information management (PHIM) activities

**Variables**	**Advanced**	**Intermediate**	**Basic**	**F**	***p***
Age	34.31 (10.70)	42.62 (13.22)	40.21 (13.27)	8.42	<0.001
Income	3.54 (1.45)	3.43 (1.45)	3.22 (1.41)	0.79	0.454
Education	4.15 (1.36)	4.22 (1.24)	4.18 (1.47)	0.05	0.954
Male	39 (57.4%)	29 (36.7%)	35 (66.0%)	12.34	0.002
Caucasian	54 (79.4%)	68 (86.1%)	47 (85.5%)	1.37	0.505
Total PHIM activity score	50.81 (6.63)	35.86 (4.51)	27.35 (5.50)	285.80	<0.001
Made appointments with my providers by email or on a website.	5.15 (1.8)	2.38 (1.60)	1.95 (1.18)	80.39	<0.001
Saw my providers use a computer or handheld device to look up test results or other information about me.	5.76 (1.59)	5.84 (1.19)	3.02 (1.97)	63.05	<0.001
Emailed my provider’s office and got an answer to my medical question.	5.32 (1.68)	2.14 (1.40)	2.04 (1.20)	111.26	<0.001
Looked for my test results on the website that my providers provided.	5.81 (1.21)	2.14 (1.53)	2.00 (1.28)	169.57	<0.001
Received a summary of my hospital visits by email or on a website.	5.66 (1.39)	1.96 (1.35)	2.02 (1.34)	164.42	<0.001
Read hospital websites about health-related information for my care.	5.62 (1.33)	4.33 (2.03)	2.84 (1.70)	39.35	<0.001
Am familiar with requesting copies of my medical records.	5.78 (1.35)	5.58 (1.27)	3.80 (1.73)	34.63	<0.001
Am familiar with searching websites to find answers to my health-related questions.	6.18 (0.81)	6.37 (0.68)	5.31 (1.59)	17.95	<0.001
Am familiar with posting/answering my health-related questions on websites.	5.53 (1.53)	5.13 (1.47)	4.38 (1.69)	8.41	<0.001

Note: Data are presented as mean (standard deviation) or frequency (%).

**Table 2:**
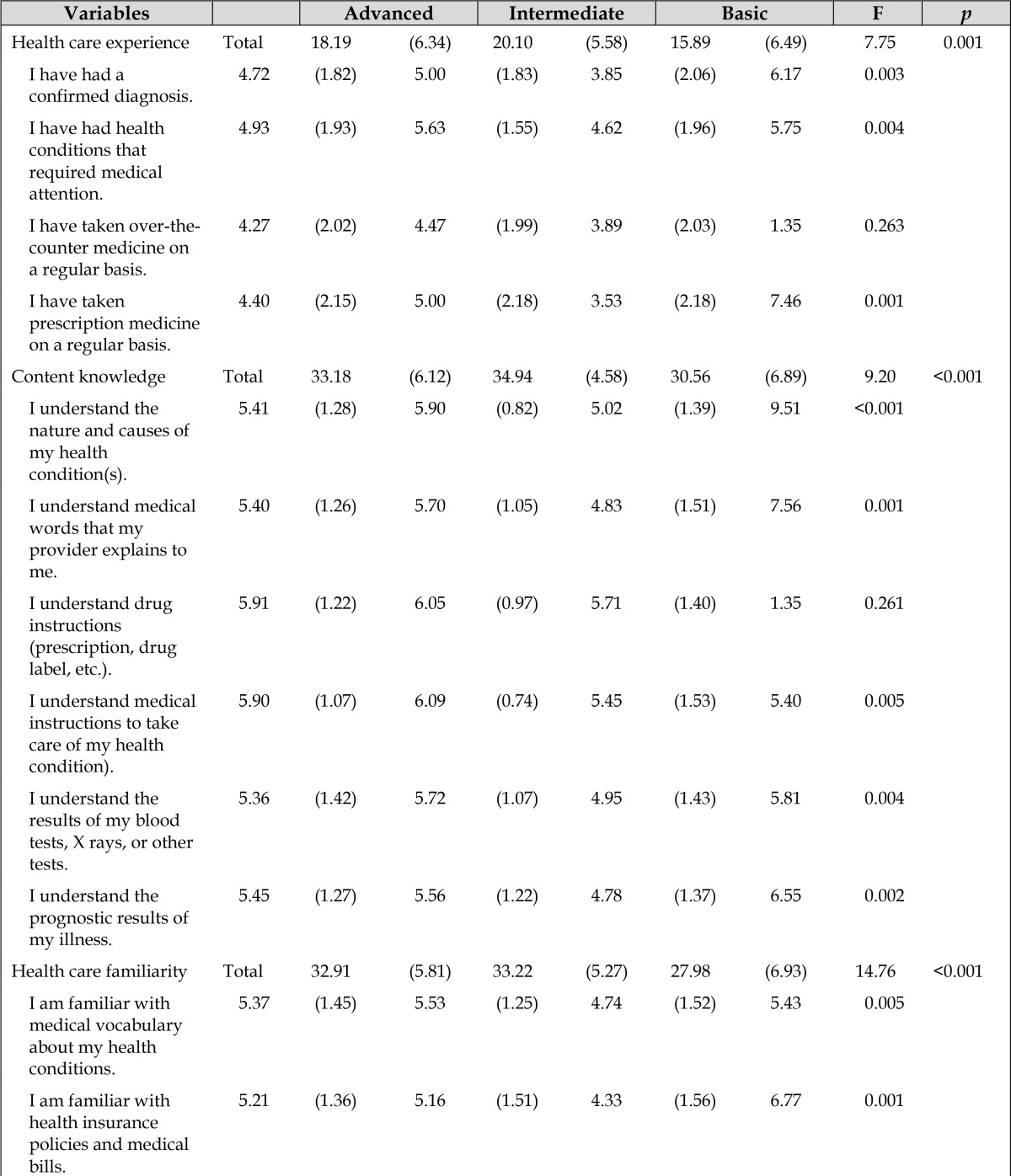

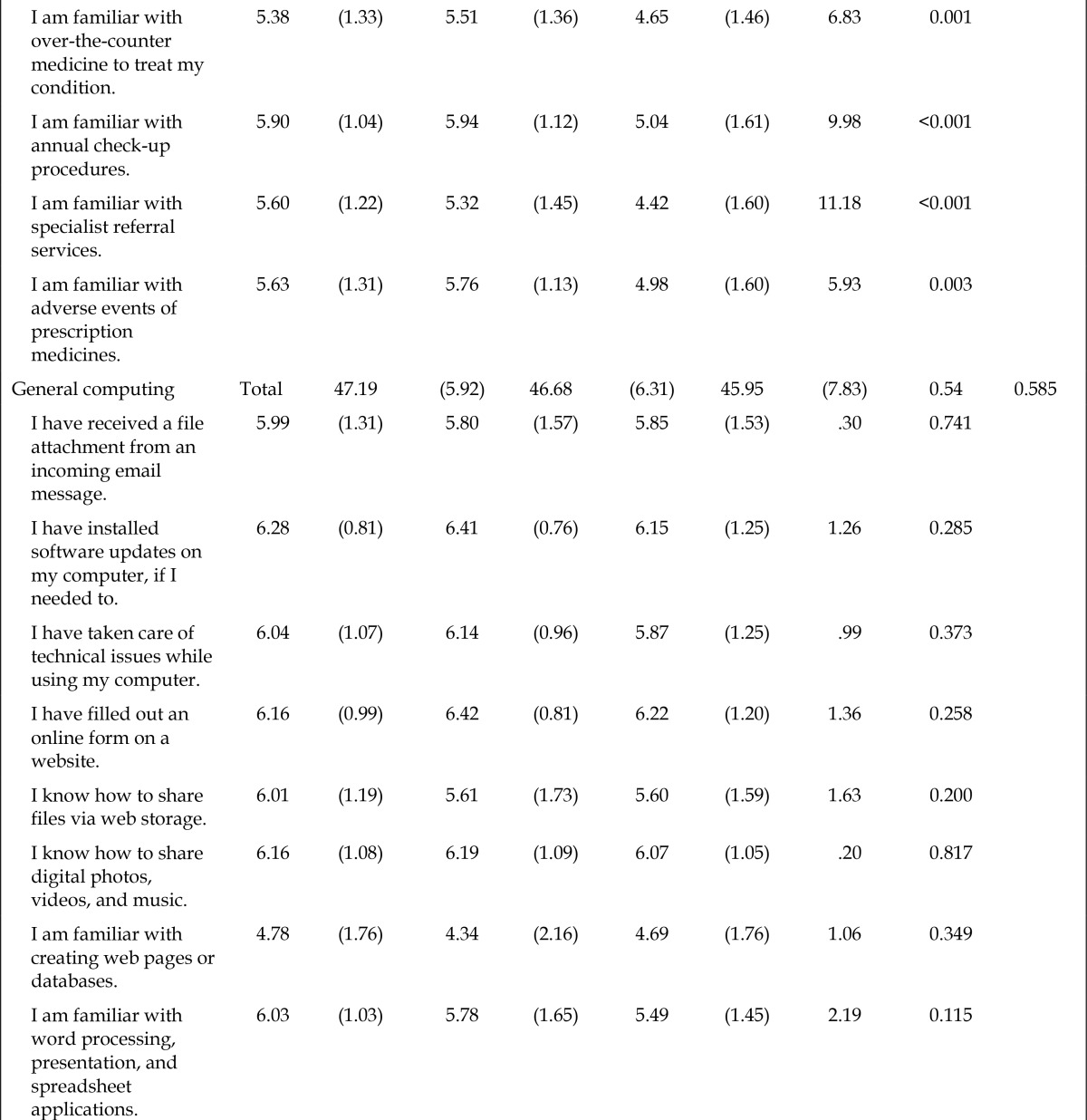
Health knowledge and technology competency

**Variables**		**Advanced**	**Intermediate**	**Basic**	**F**	***p***
Health care experience	Total	18.19 (6.34)	20.10 (5.58)	15.89 (6.49)	7.75	0.001
I have had a confirmed diagnosis.		4.72 (1.82)	5.00 (1.83)	3.85 (2.06)	6.17	0.003
I have had health conditions that required medical attention.		4.93 (1.93)	5.63 (1.55)	4.62 (1.96)	5.75	0.004
I have taken over-the-counter medicine on a regular basis.		4.27 (2.02)	4.47 (1.99)	3.89 (2.03)	1.35	0.263
I have taken prescription medicine on a regular basis.		4.40 (2.15)	5.00 (2.18)	3.53 (2.18)	7.46	0.001
Content knowledge	Total	33.18 (6.12)	34.94 (4.58)	30.56 (6.89)	9.20	<0.001
I understand the nature and causes of my health condition(s).		5.41 (1.28)	5.90 (0.82)	5.02 (1.39)	9.51	<0.001
I understand medical words that my provider explains to me.		5.40 (1.26)	5.70 (1.05)	4.83 (1.51)	7.56	0.001
I understand drug instructions (prescription, drug label, etc.).		5.91 (1.22)	6.05 (0.97)	5.71 (1.40)	1.35	0.261
I understand medical instructions to take care of my health condition).		5.90 (1.07)	6.09 (0.74)	5.45 (1.53)	5.40	0.005
I understand the results of my blood tests, X rays, or other tests.		5.36 (1.42)	5.72 (1.07)	4.95 (1.43)	5.81	0.004
I understand the prognostic results of my illness.		5.45 (1.27)	5.56 (1.22)	4.78 (1.37)	6.55	0.002
Health care familiarity	Total	32.91 (5.81)	33.22 (5.27)	27.98 (6.93)	14.76	<0.001
I am familiar with medical vocabulary about my health conditions.		5.37 (1.45)	5.53 (1.25)	4.74 (1.52)	5.43	0.005
I am familiar with health insurance policies and medical bills.		5.21 (1.36)	5.16 (1.51)	4.33 (1.56)	6.77	0.001
I am familiar with over-the-counter medicine to treat my condition.		5.38 (1.33)	5.51 (1.36)	4.65 (1.46)	6.83	0.001
I am familiar with annual check-up procedures.		5.90 (1.04)	5.94 (1.12)	5.04 (1.61)	9.98	<0.001
I am familiar with specialist referral services.		5.60 (1.22)	5.32 (1.45)	4.42 (1.60)	11.18	<0.001
I am familiar with adverse events of prescription medicines.		5.63 (1.31)	5.76 (1.13)	4.98 (1.60)	5.93	0.003
General computing	Total	47.19 (5.92)	46.68 (6.31)	45.95 (7.83)	0.54	0.585
I have received a file attachment from an incoming email message.		5.99 (1.31)	5.80 (1.57)	5.85 (1.53)	.30	0.741
I have installed software updates on my computer, if I needed to.		6.28 (0.81)	6.41 (0.76)	6.15 (1.25)	1.26	0.285
I have taken care of technical issues while using my computer.		6.04 (1.07)	6.14 (0.96)	5.87 (1.25)	.99	0.373
I have filled out an online form on a website.		6.16 (0.99)	6.42 (0.81)	6.22 (1.20)	1.36	0.258
I know how to share files via web storage.		6.01 (1.19)	5.61 (1.73)	5.60 (1.59)	1.63	0.200
I know how to share digital photos, videos, and music.		6.16 (1.08)	6.19 (1.09)	6.07 (1.05)	.20	0.817
I am familiar with creating web pages or databases.		4.78 (1.76)	4.34 (2.16)	4.69 (1.76)	1.06	0.349
I am familiar with word processing, presentation, and spreadsheet applications.		6.03 (1.03)	5.78 (1.65)	5.49 (1.45)	2.19	0.115
Information management and Internet use	Total	60.75 (7.15)	60.92 (5.63)	57.38 (8.55)	4.86	0.013
I know how to find medical information, if I need to.		6.15 (0.93)	6.18 (0.98)	5.89 (1.09)	1.51	0.224
I know how to scan or save my medical information.		5.88 (1.16)	5.68 (1.47)	5.49 (1.29)	1.31	0.272
I am familiar with tagging keywords for photos or videos.		5.84 (1.30)	5.53 (1.60)	5.24 (1.63)	2.42	0.092
I am familiar with uploading and downloading my information.		6.13 (0.83)	6.19 (0.95)	5.91 (1.25)	1.34	0.263
I am familiar with which health information should be kept or removed.		5.57 (1.32)	4.73 (1.74)	4.95 (1.43)	5.78	0.004
I am familiar with evaluating the health resources I find on the Internet.		5.88 (1.00)	6.01 (0.87)	5.42 (1.10)	6.26	0.002
I know how to use a computer to check news, weather, or sports.		6.31 (0.99)	6.73 (0.47)	6.24 (1.44)	5.18	0.006
I know how to use a computer for participating in social media sites.		6.31 (1.03)	6.51 (0.89)	6.11 (1.41)	2.15	0.120
I know how to use a computer for banking, paying bills, or shopping.		6.43 (0.80)	6.68 (0.59)	6.22 (1.27)	4.50	0.012
I know how to use a computer for searching for information.		6.43 (0.82)	6.75 (0.47)	6.15 (1.48)	6.60	0.002

Note: Data are presented as mean (standard deviation).

**Table 3:**
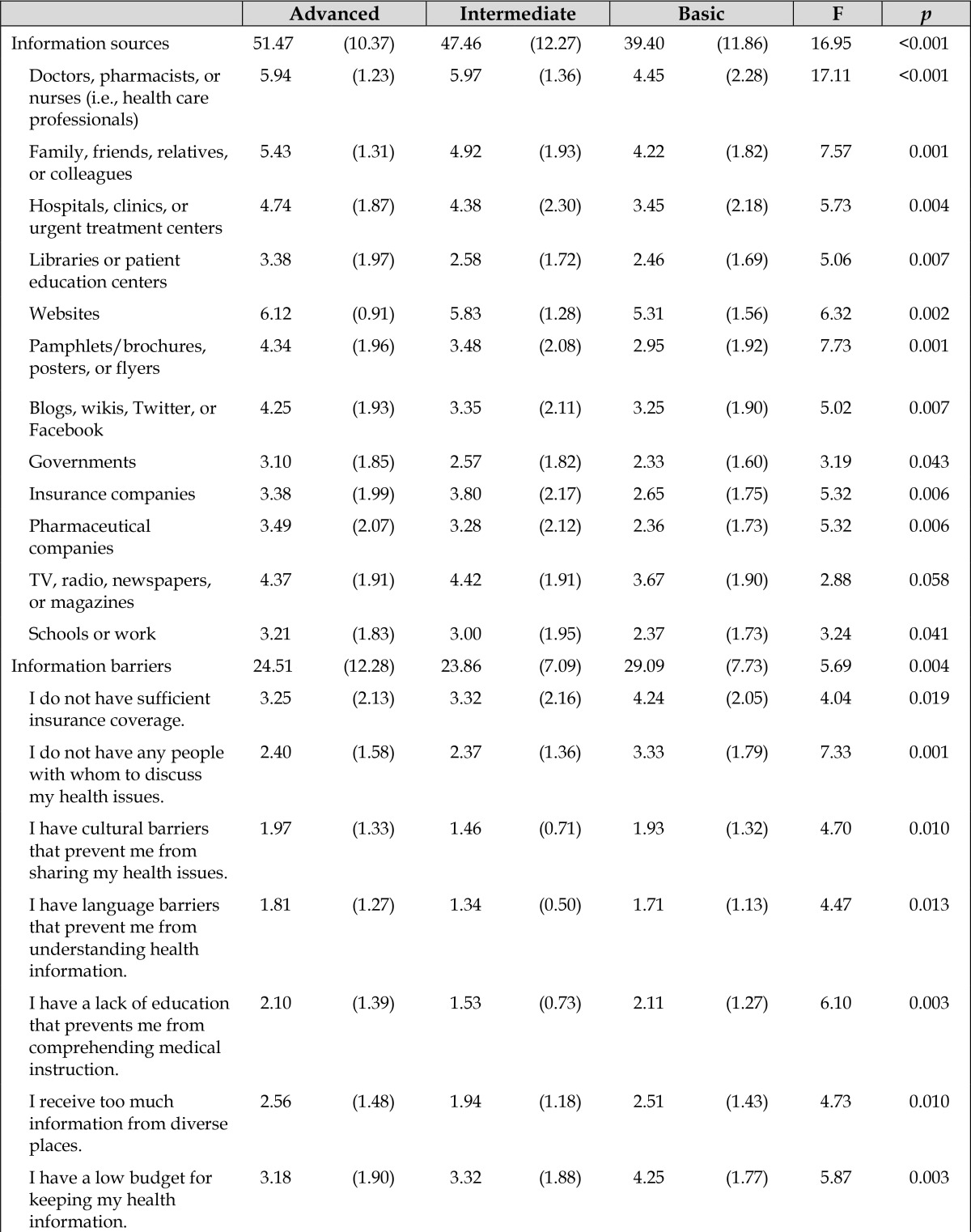
Health information sources and barriers

	**Advanced**	**Intermediate**	**Basic**	**F**	***p***
Information sources	51.47 (10.37)	47.46 (12.27)	39.40 (11.86)	16.95	<0.001
Doctors, pharmacists, or nurses (i.e., health care professionals)	5.94 (1.23)	5.97 (1.36)	4.45 (2.28)	17.11	<0.001
Family, friends, relatives, or colleagues	5.43 (1.31)	4.92 (1.93)	4.22 (1.82)	7.57	0.001
Hospitals, clinics, or urgent treatment centers	4.74 (1.87)	4.38 (2.30)	3.45 (2.18)	5.73	0.004
Libraries or patient education centers	3.38 (1.97)	2.58 (1.72)	2.46 (1.69)	5.06	0.007
Websites	6.12 (0.91)	5.83 (1.28)	5.31 (1.56)	6.32	0.002
Pamphlets/brochures, posters, or flyers	4.34 (1.96)	3.48 (2.08)	2.95 (1.92)	7.73	0.001
Blogs, wikis, Twitter, or Facebook	4.25 (1.93)	3.35 (2.11)	3.25 (1.90)	5.02	0.007
Governments	3.10 (1.85)	2.57 (1.82)	2.33 (1.60)	3.19	0.043
Insurance companies	3.38 (1.99)	3.80 (2.17)	2.65 (1.75)	5.32	0.006
Pharmaceutical companies	3.49 (2.07)	3.28 (2.12)	2.36 (1.73)	5.32	0.006
TV, radio, newspapers, or magazines	4.37 (1.91)	4.42 (1.91)	3.67 (1.90)	2.88	0.058
Schools or work	3.21 (1.83)	3.00 (1.95)	2.37 (1.73)	3.24	0.041
Information barriers	24.51 (12.28)	23.86 (7.09)	29.09 (7.73)	5.69	0.004
I do not have sufficient insurance coverage.	3.25 (2.13)	3.32 (2.16)	4.24 (2.05)	4.04	0.019
I do not have any people with whom to discuss my health issues.	2.40 (1.58)	2.37 (1.36)	3.33 (1.79)	7.33	0.001
I have cultural barriers that prevent me from sharing my health issues.	1.97 (1.33)	1.46 (0.71)	1.93 (1.32)	4.70	0.010
I have language barriers that prevent me from understanding health information.	1.81 (1.27)	1.34 (0.50)	1.71 (1.13)	4.47	0.013
I have a lack of education that prevents me from comprehending medical instruction.	2.10 (1.39)	1.53 (0.73)	2.11 (1.27)	6.10	0.003
I receive too much information from diverse places.	2.56 (1.48)	1.94 (1.18)	2.51 (1.43)	4.73	0.010
I have a low budget for keeping my health information.	3.18 (1.90)	3.32 (1.88)	4.25 (1.77)	5.87	0.003
I have a low budget for keeping my health information.	3.18 (1.90)	3.32 (1.88)	4.25 (1.77)	5.87	0.003
I have technology barriers that prevent me from searching for health information.	2.16 (1.53)	1.66 (0.75)	1.98 (1.21)	3.41	0.035
My providers do not offer electronic copies of my health records.	2.51 (1.54)	4.10 (2.02)	4.11 (1.63)	18.19	<0.001
I do not have enough time to organize my medical records.	2.76 (1.70)	2.86 (1.60)	3.00 (1.40)	0.34	0.715

Note: Data are presented as mean (standard deviation).

### Demographic characteristics and personal health information management (PHIM) activities

Three groups of individuals were formed based on their familiarity with PHIM activities: Advanced (n=68, 34%), Intermediate (n=79, 39.5%), and Basic (n=55, 26.5%) (Table 1). There were significant differences between groups in overall PHIM activity score as well as all individual PHIM activity scores as determined by one-way ANOVAs. In general, post-hoc tests showed that the Advanced group was more likely to engage in provider- or hospital-initiated PHIM activities such as emailing with providers, viewing test results online, and receiving summaries of hospital visits via email or website than the Intermediate and Basic groups (*p*<0.05). However, there were no significant differences between the Intermediate and Basic groups in these activities (*p*>0.05). Also, Advanced and Intermediate groups were more likely to engage in individual-initiated activities such as searching websites for health information and posting or answering health-related questions on websites than the Basic group (*p*<0.05), whereas there were no significant differences between Advanced and Intermediate groups (*p*>0.05).

Among the demographic variables that were analyzed, age and gender significantly distinguished the three groups, whereas race, education, and income did not. The Advanced group was the youngest and the Basic group contained the highest proportion of males, whereas the Intermediate group was the oldest and contained the lowest proportion of males.

### Health knowledge and technology competency

We next examined differences among the 3 PHIM groups in their health knowledge and technology competency (Table 2). We found significant differences among groups in all 3 health knowledge domains as determined by one-way ANOVA. In general, post-hoc tests showed that the Intermediate group had significantly higher health care experience and content knowledge than the Advanced and Basic groups (*p*<0.05). We found no significant difference among the 3 groups in general computing (*p*>0.05), but the difference among groups in information management and Internet use was statistically significant (*p*<0.05). Post-hoc tests showed that the Basic group had significantly lower information management skills and Internet use than the Advanced and Intermediate groups (*p*<0.05).

### Information sources and barriers

We also examined differences among the 3 PHIM groups in their information sources and barriers (Table 3). We found significant differences among groups in the total information source score and most individual information sources as determined by one-way ANOVAs. In general, post-hoc tests revealed significant differences in individual information sources between Advanced and Basic groups and between Intermediate and Basic groups (*p*<0.05), whereas there were no differences between Advanced and Intermediate groups (*p*>0.05). Information sources such as websites or health care professionals were most frequently used, whereas institution- or organization-based information sources—such as those from governments, schools, work, libraries, or patient education centers—were less likely to be used.

We also found significant differences among groups in total information barrier score and most individual information barriers as determined by one-way ANOVAs. Post-hoc tests revealed significant differences between Advanced and Basic groups in four issues: “I do not have sufficient insurance coverage”; “I do not have any people with whom to discuss my health issues”; “I have a low budget for keeping my health information”; and “My providers do not offer electronic copies of my health records” (*p*<0.05).

### Self-reported general literacy and objectively measured health literacy

For analysis of health literacy, we included 152 survey respondents (75.25%) out of 202 total survey respondents who answered and took the 11 general literacy questions and the RTI literacy test (Table 4). Interestingly, the Advanced group self-reported the highest general literacy but scored the lowest on an objective health literacy test. We found a significant difference among groups in self-reported aspects of general literacy as determined by one-way ANOVAs. Post-hoc tests showed that the Basic group self-reported lower overall general literacy than the Intermediate or Advanced groups (*p*<0.05), whereas there was no difference between Intermediate and Advanced groups (*p*>0.05). Concerning individual self-reported general literacy items, post-hoc tests generally showed significant differences between the Advanced and Basic groups (*p*<0.05).

**Table 4:** Self-reported and Research Triangle Initiative (RTI) health literacy test scores

**Variables**	**Advanced**	**Intermediate**	**Basic**	**F**	***p***
Overall self-reported general literacy	67.49 (6.97)	64.00 (8.11)	59.34 (9.68)	10.95	<0.001
Reading comprehension	6.39 (0.67)	6.29 (0.76)	5.78 (1.24)	5.97	0.003
Numerical computation	6.02 (1.18)	5.47 (1.16)	5.32 (1.31)	4.47	0.013
Oral communication	6.16 (1.01)	5.74 (1.17)	5.34 (1.39)	5.38	0.006
Visual interpretation	6.31 (0.87)	6.13 (0.84)	5.76 (1.11)	4.02	0.020
Internet searching	6.41 (0.73)	6.37 (0.81)	5.98 (1.06)	3.42	0.035
Database searching	6.02 (1.01)	5.77 (1.06)	5.12 (1.23)	7.94	0.001
Mobile applications	5.92 (1.05)	5.38 (1.59)	4.85 (1.53)	6.20	0.003
Writing	6.21 (0.87)	5.89 (1.03)	5.48 (1.15)	5.67	0.004
Speaking	6.27 (0.91)	5.92 (1.01)	5.51 (1.34)	5.41	0.005
Listening	6.43 (0.74)	6.13 (0.98)	5.78 (1.08)	5.29	0.006
Negotiation	5.61 (1.17)	5.00 (1.53)	4.56 (1.34)	6.74	0.002
Overall RTI test score	18.09 (5.05)	20.56 (2.45)	18.78 (5.01)	4.50	0.013
Print-Prose	4.14 (1.11)	4.57 (0.66)	4.37 (0.97)	2.78	0.066
Print-Document	5.77 (2.19)	6.87 (1.12)	5.98 (2.12)	5.11	0.007
Print-Quantitative	2.98 (0.94)	3.19 (0.59)	3.02 (1.01)	0.83	0.440
Oral	3.12 (1.03)	3.48 (0.72)	3.05 (1.09)	2.99	0.054
Internet	2.28 (0.83)	2.44 (0.86)	2.37 (0.97)	0.42	0.658

Note: Data are presented as mean (standard deviation).

The RTI health literacy test was administered to objectively measure health literacy. We found a significant difference among groups in overall RTI test score, with post-hoc tests showing that the Intermediate group scored higher than the Basic and Advanced groups (*p*<0.05). Considering the 5 subscales, post-hoc tests revealed that the Intermediate group scored higher than the Advanced group on the print-document subscale (*p*<0.05), whereas there were no group differences for the other subscale items. We then computed correlation coefficients between self-reported general literacy and RTI subscale scores. We found only some weak correlations between self-reported general literacy and objective PHIM literacy (Table 5).

**Table 5:** Correlations between self-rated and RTI health literacy test subscale scores

**Self-rated literacy**	**Print-prose**	**Print-document**	**Print-quantitative**	**Oral**	**Internet**
Reading comprehension					
Correlation	**0.190**	**0.233**	**0.190**	0.139	**0.197**
*p*	0.025	0.006	0.026	0.105	0.020
Numerical computation					
Correlation	0.149	0.140	0.159	0.099	0.123
*p*	0.080	0.100	0.063	0.246	0.150
Oral communication					
Correlation	0.113	0.115	0.026	−0.049	0.068
*p*	0.184	0.178	0.758	0.567	0.430
Visual interpretation					
Correlation	**0.184**	**0.186**	0.095	0.088	**0.199**
Sig.	0.030	0.029	0.269	0.307	0.019
Internet searching					
Correlation	**0.219**	**0.308**	**0.226**	**0.241**	**0.237**
*p*	0.010	0.000	0.008	0.004	0.005
Database searching					
Correlation	0.088	0.097	0.087	0.100	0.163
*p*	0.303	0.255	0.311	0.245	0.057
Mobile applications					
Correlation	−0.052	0.020	0.035	0.090	0.150
*p*	0.544	0.815	0.688	0.298	0.081
Writing					
Correlation	0.066	0.125	0.087	0.047	0.**180**
*p*	0.444	0.145	0.313	0.587	0.036
Speaking					
Correlation	0.094	0.156	0.101	0.086	0.087
*p*	0.272	0.066	0.241	0.314	0.310
Listening					
Correlation	**0.176**	**0.256**	0.132	**0.175**	**0.199**
*p*	0.038	0.002	0.123	0.040	0.020
Negotiation					
Correlation	−0.078	−0.139	−0.016	−0.149	−0.102
*p*	0.363	0.103	0.850	0.080	0.233

Note: Statistically significant (*p*<0.05) correlations are shown in bold.

## DISCUSSION

In this study, we used the K-means clustering technique to form three distinct PHIM groups—Advanced, Intermediate, and Basic—based on participants’ familiarity with nine distinct PHIM activities, and we characterized the groups in terms of their demographics, health knowledge, technology competency, and information sources and barriers. Furthermore, we examined differences among PHIM groups in self-rated and objective test-based health literacy.

We formed three PHIM groups based on their familiarity with PHIM-related activities. The formation of three groups is a common approach when using health literacy instruments to assess an individual’s capability in managing health-related activities [26, 27]. We confirmed that the three PHIM groups as formed by K-means clustering showed significant differences in their PHIM-related activities. Interestingly, participants in the Advanced group gave high ratings for institution-supported PHIM experiences, whereas those in the Intermediate and Basic groups gave low ratings for these activities (i.e., emailing with providers, accessing test results online, making appointments online). These activities are only possible through provider-initiated PHR applications, or so-called tethered PHR applications, which are connected to hospitals’ EHR systems.

The 2009 US Health Information Technology for Economic and Clinical Health Act created a meaningful use incentive program to provide financial support to health care providers and health systems adopting EHR technologies, which has significantly increased PHR adoption [28]. With the meaningful use program, more hospitals provide access to patient portals containing comprehensive medical records. Thus, patients (or their caregivers) with hospital-supported PHR access will have more opportunities to improve their PHIM skills than those without access to such systems. Although not a direct implication of our study findings, our results could indirectly imply that untethered, consumer-driven patient portals are not sufficient unless hospitals (or physicians’ offices) input their records into these patient portals.

We found that the three PHIM groups showed distinct demographic profiles in terms of age and gender, whereas the other demographic characteristics were similar across groups. The demographic profiles associated with different levels of health literacy are not yet conclusive. In a recent comprehensive review, the Agency for Healthcare Research and Quality reports the existence of ethnicity-based health literacy disparities [274], yet we found no significant differences in ethnicity among different PHIM groups, perhaps due to the fact that our sample was overwhelmingly Caucasian. Therefore, health literacy disparities among distinct demographic groups should be further studied for stronger evidence of existing links.

We found that the Intermediate group was highly knowledgeable, the most experienced, and relatively familiar with health information content and health care services. This result was somewhat related to an earlier finding that the Intermediate group was the oldest and most educated [29]. Consistent with previous literature reporting that older adults are more likely to have health issues and more experience with managing chronic health conditions than younger adults [30], we found that the older adults in the Intermediate group showed the greatest knowledge, experience, and familiarity with various health activities and health care services. However, the Intermediate group did not exhibit the highest level of familiarity with PHIM activities. In other words, if someone is highly competent in health issues, this does not guarantee a high level of PHIM literacy.

Additionally, we assessed differences among the three PHIM groups in general computing and Internet activities. We found no significant differences among groups in general computing skills. However, the Intermediate group scored slightly higher than the other two groups on certain technology-related Internet activities, such as evaluating health resources found on the Internet; using a computer to check news, weather, or sports; using a computer for banking, paying bills, or shopping; and using a computer to search for information. Considering the differences in age between groups, our results are consistent with those of Pak and colleagues, who found that older people are more likely to adopt some forms of technology than younger people [31].

Not surprisingly, the reported information sources and barriers were consistent with health and technology knowledge-related ratings. In other words, individuals in the Advanced PHIM group reported more exposure to information sources and fewer information barriers. Interestingly, the most popular information sources across the three groups were websites, followed by health care professionals. However, information provided by libraries and patient education centers were not fully utilized by the study participants. Therefore, a new approach to expanding medical libraries or patient education centers is needed. For example, Huber and colleagues proposed expanding a patient navigator program by including library and information science professionals who are “tasked with the selection and dissemination of understandable, relevant, culturally appropriate information.” Likewise, medical librarians could work with PHR teams or patient navigator teams to help solicit relevant personal health documents from patients, perform as mediated searchers, and train users of PHR systems [32, 33].

The Advanced PHIM group gave the highest self-ratings for all eleven general literacy scales. However, the Advanced group scored lowest on the objective RTI health literacy test, whereas the Intermediate group scored the highest. This finding implies that individuals who are relatively experienced and more knowledgeable about health care have greater health literacy, whereas those who rate themselves highly in PHIM-related activities have lower health literacy. In other words, perhaps younger individuals have more confidence with technology-related tasks but lack health knowledge and experience. Among the five RTI subscales, most participants scored low in Internet searching and quantitative literacy. Likewise, in a previous health literacy study, numeracy was reported as most problematic and challenging compared with other literacy skills [29].

In addition, all three PHIM groups self-rated their Internet searching skills highly but scored lowest on the Internet subscale of the RTI test. Based on the mean subscale scores, most participants did not answer half of the Internet-related questions correctly. Considering the high reliance on Internet searching skills due to current Internet-based PHR applications, Internet literacy should be improved to meet competency requirements for the effective use of PHR applications. Also, Internet-related literacy should be included in PHR literacy scales and should be an important consideration with developing personalized PHR training programs.

Our study has some limitations. First, the recruitment of participants from a crowd-sourced Internet service might introduce sampling bias, especially as these heavy Internet users might not accurately reflect the general public and their level of health literacy. Second, although the RTI test objectively measures diverse aspects of health literacy, it does not contain PHIM-specific measurement items. Multiple aspects of health literacy items covering not only health knowledge, but also diverse information management activities should be considered in future PHIM literacy or PHR usability studies [34].
